# Supplementary material for: Recombinant Protein Vaccines Formulated with Enantio-Specific Cationic Lipid R-DOTAP Induce Protective Cellular and Antibody-Mediated Immune Responses in Mice
Source: Viruses. 2023 Feb 4;15(2):432. doi: 10.3390/v15020432 (PMC9965888; doi:10.3390/v15020432)
Supplement: Supplementary file 1 [file viruses-15-00432-s001.zip › viruses-2147397-supplementary.pdf]

Supplementary Table S1: Particle size, polydispersity index (PDI) and zeta potential of R-DOTAP formulations.

**Formulation**	**Average particle Dia. (nm) ± Stdev**	**PDI Average ± Stdev**	**ζ Potential (mv) average ± Stdev**
R-DOTAP	152.87±0.40	0.10± 0.01	54.7 ± 2.10
COBRA-Y2. R-DOTAP	148.67±0.70	0.13± 0.01	45.90±1.82
COBRA-NG2.R-DOTAP	147.90±1.05	0.11± 0.01	46.70±1.31
COBRA-Y2.NG2. R-DOTAP	148.2± 1.13	0.11± 0.00	46.20±0.10

Supplementary Figure S1

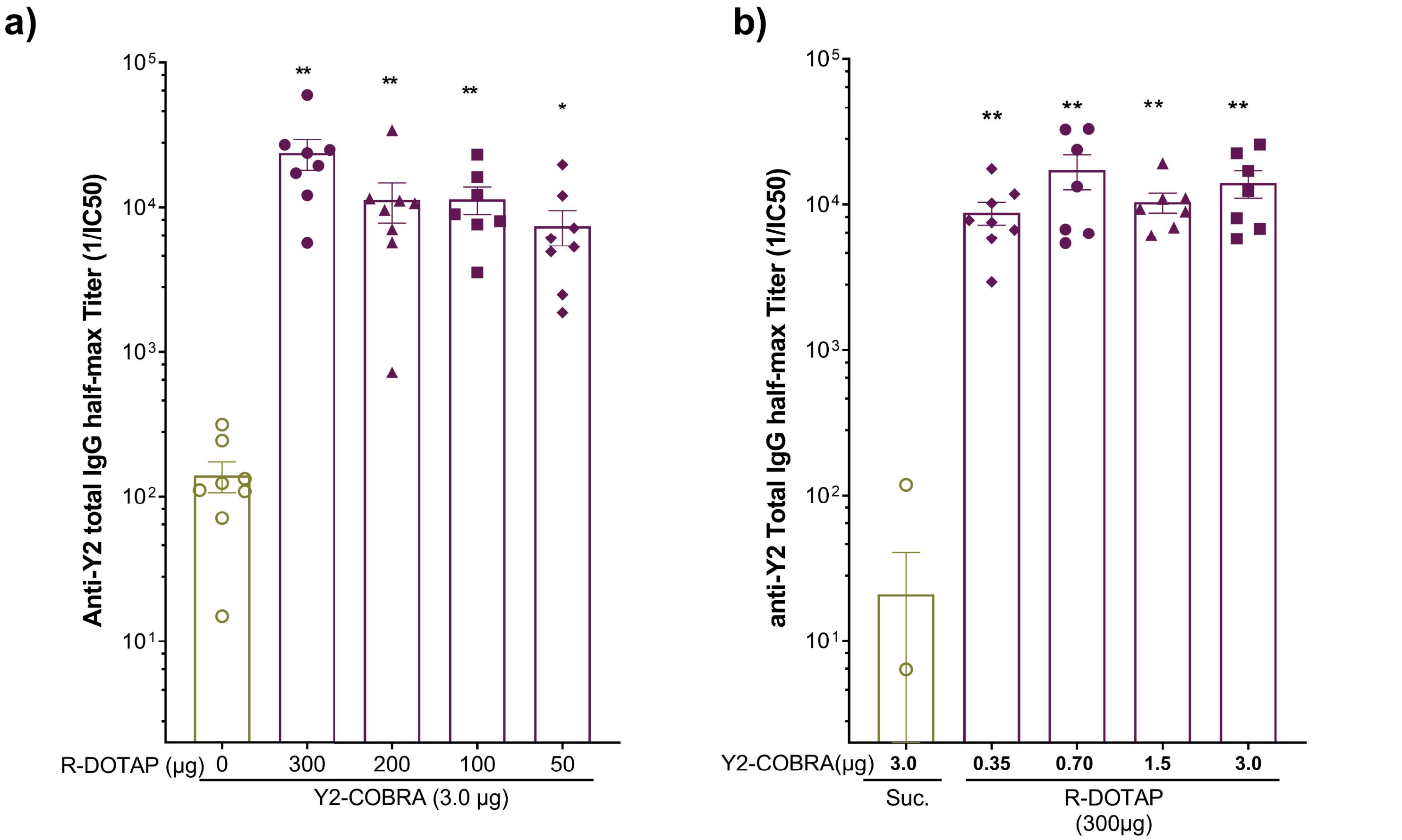

Supplement Figure S1: R-DOTAP is effective over a wide dose range and mediates dose-sparing of COBRA antigens. Groups of BALB/cJ (n=6-8) mice were immunized on day 0 and day 21 with 3µg of COBRA-Y2 protein and varying doses of R-DOTAP (a), or a 300 µg of R-DOTAP and varying doses of COBRA-Y2 protein or sucrose (Suc.) (b). Serum samples obtained from vaccinated mice on day 35 (14 days after the second dose) were measured for anti-COBRA-Y2 total IgG antibody by ELISA. Data represent mean ± SEM of half-max titers from each mouse. Comparisons between sucrose alone or R-DOTAP groups was performed using Student’s t-test (unpaired-two tailed).** P < 0.05

Supplementary Figure S2

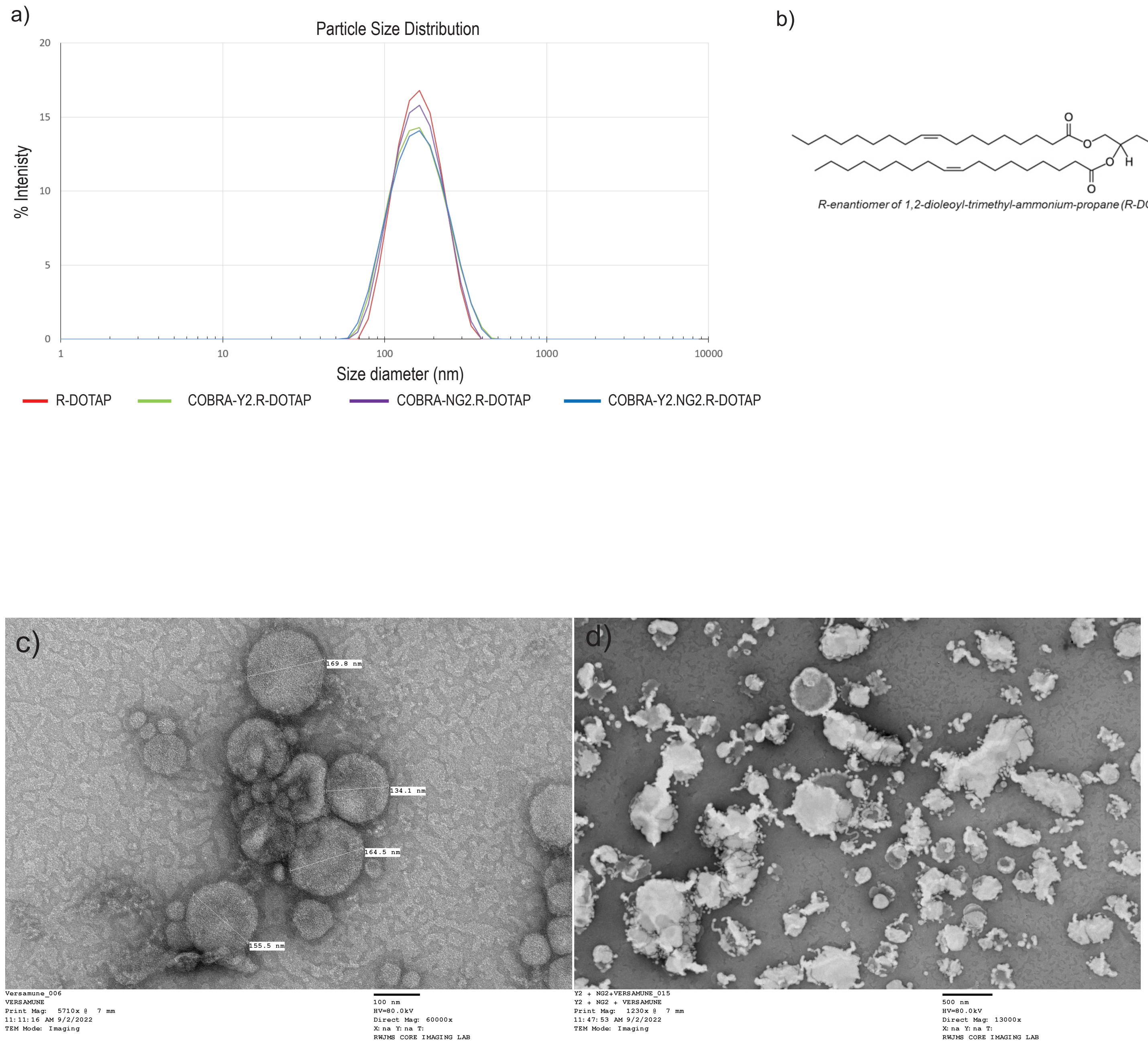

Supplementary Figure S2: Average particle size (a) for R-DOTAP liposomal nanoparticles and vaccine formulations containing R-DOTAP and protein antigens (COBRA-Y2, or COBRA-NG2 , or both) were measured under indicated buffer conditions. Data presented are representative histograms of three independent measurements. b) Chemical structure of cationic lipid R-DOTAP (1,2-Dioleoyl-3-trimethylammonium propane). c-d) Representative TEM images of R-DOTAP nanoparticles or vaccine formulation (COBRA-Y2.NG2.R-DOTAP) contaning R-DOTAP (c) and COBRA proteins mixtures (d).

Supplementary Figure S3 a)

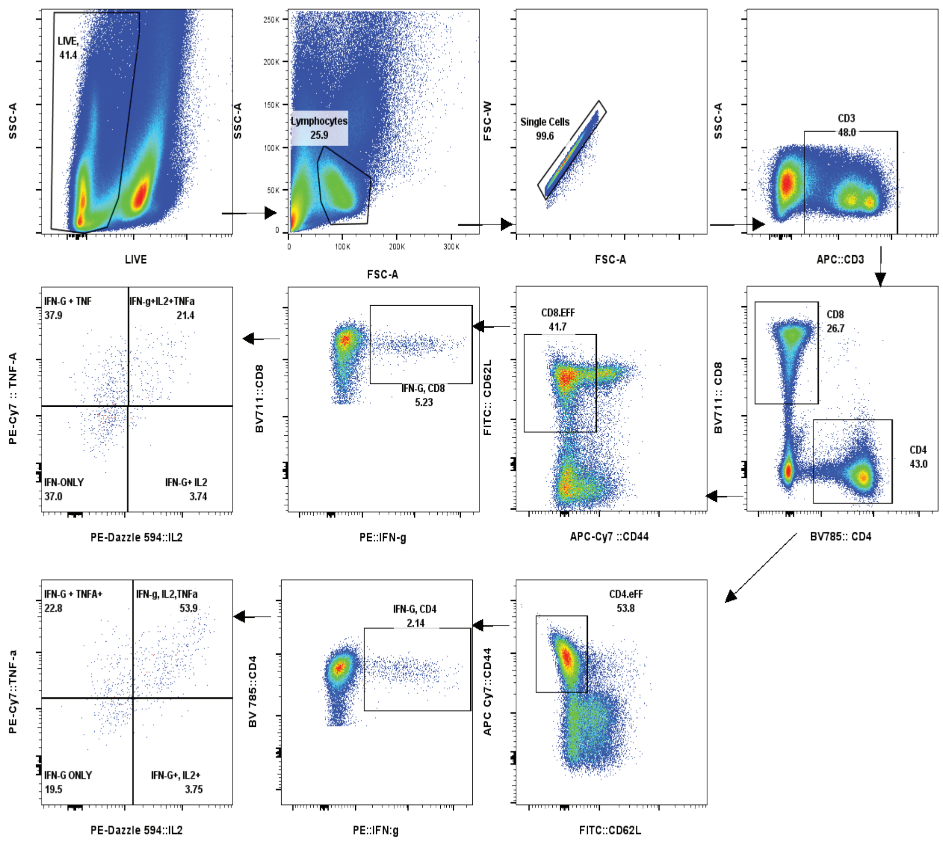

b)

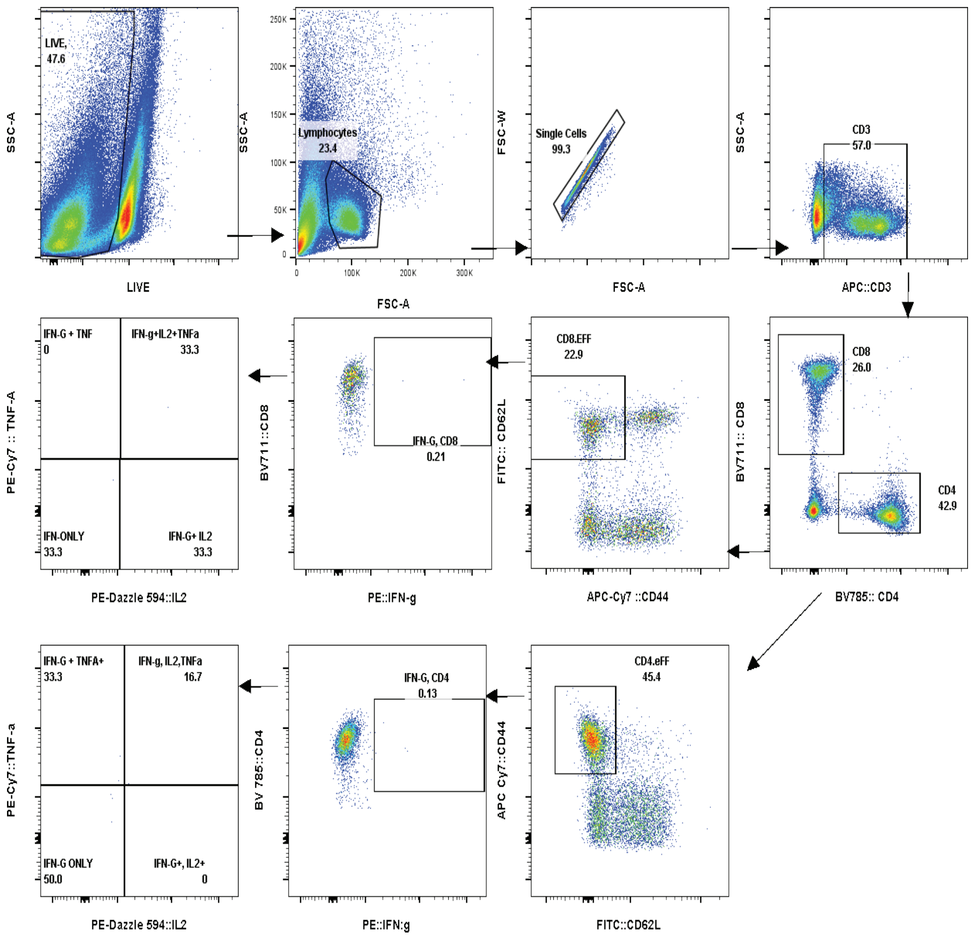

Supplement Figure S3: a) Representative flow gating scheme for figure 5. All gates for polyfunctional cytokine producing cells were drawn using fluorescence-minus-one controls to verify the lack of non-specific binding or bleed over from other antibodies/fluorochromes. Arrows indicate the gating strategy. CD44hi and CD62L low cells among CD4 and CD8 T cells were identified as effector cells. Polyfunctional cells in effector cells were gated first to identify IFN-gamma-producing cells and then separated into the four cytokine groups. b) The specificity of the cytokine production was further verified by staining un-stimulated cells which show no significant IFN gamma, IL-2, or TNFα staining. Gating on naïve cells also showed a lack of cytokine staining (not shown).
